# A Calcium-Dependent Plasticity Rule for HCN Channels Maintains Activity Homeostasis and Stable Synaptic Learning

**DOI:** 10.1371/journal.pone.0055590

**Published:** 2013-02-04

**Authors:** Suraj Honnuraiah, Rishikesh Narayanan

**Affiliations:** Cellular Neurophysiology Laboratory, Molecular Biophysics Unit, Indian Institute of Science, Bangalore, India; Tokai University, Japan

## Abstract

Theoretical and computational frameworks for synaptic plasticity and learning have a long and cherished history, with few parallels within the well-established literature for plasticity of voltage-gated ion channels. In this study, we derive rules for plasticity in the hyperpolarization-activated cyclic nucleotide-gated (HCN) channels, and assess the synergy between synaptic and HCN channel plasticity in establishing stability during synaptic learning. To do this, we employ a conductance-based model for the hippocampal pyramidal neuron, and incorporate synaptic plasticity through the well-established Bienenstock-Cooper-Munro (BCM)-like rule for synaptic plasticity, wherein the direction and strength of the plasticity is dependent on the concentration of calcium influx. Under this framework, we derive a rule for HCN channel plasticity to establish homeostasis in synaptically-driven firing rate, and incorporate such plasticity into our model. In demonstrating that this rule for HCN channel plasticity helps maintain firing rate homeostasis after bidirectional synaptic plasticity, we observe a linear relationship between synaptic plasticity and HCN channel plasticity for maintaining firing rate homeostasis. Motivated by this linear relationship, we derive a calcium-dependent rule for HCN-channel plasticity, and demonstrate that firing rate homeostasis is maintained in the face of synaptic plasticity when moderate and high levels of cytosolic calcium influx induced depression and potentiation of the HCN-channel conductance, respectively. Additionally, we show that such synergy between synaptic and HCN-channel plasticity enhances the stability of synaptic learning through metaplasticity in the BCM-like synaptic plasticity profile. Finally, we demonstrate that the synergistic interaction between synaptic and HCN-channel plasticity preserves robustness of information transfer across the neuron under a rate-coding schema. Our results establish specific physiological roles for experimentally observed plasticity in HCN channels accompanying synaptic plasticity in hippocampal neurons, and uncover potential links between HCN-channel plasticity and calcium influx, dynamic gain control and stable synaptic learning.

## Introduction

Theoretical and computational frameworks for synaptic plasticity have a long and cherished history, with proven utilities ranging from understanding the underlying biophysical and biochemical mechanisms to solving complex engineering problems [1]–[8]. A central question in synaptic learning systems is on how they retain their ability to learn in the future and maintain stability, in the face of the adaptations that they undergo during the learning process. A prominent postulate, with a large body of experimental and theoretical evidence in support, is that neural systems accomplish such stability through concurrent regulatory mechanisms that recruit plasticity in synaptic and/or intrinsic neuronal properties [1], [9]–[17]. Whereas several computational approaches have been helpful in enhancing our understanding of synaptically mediated mechanisms for stable learning [1], [7], [14], [17]–[21], mechanisms for stable synaptic learning mediated by intrinsic plasticity have not been studied in quantitative detail. Furthermore, biophysically and biochemically rooted plasticity rules, which have been demonstrably elucidative in the synaptic plasticity literature [3], [4], [6], [17], [22], [23], have no counterparts in the intrinsic plasticity literature, thus contributing to the lacuna in models for stability through intrinsic plasticity.

The hyperpolarization-activated, cyclic nucleotide-gated (HCN) channels, that mediate the hyperpolarization-activated *h* current, have been postulated as a prominent mechanism that can mediate activity homeostasis in hippocampal neurons [13], [24]–[27]. In this study, we quantitatively examine the validity of this postulate employing conductance-based models and biophysically rooted plasticity rules for the *h* conductance. In doing this, we first posed the question on what changes in the *h* conductance would be required for it to maintain activity homeostasis, when perturbed by a calcium-dependent bidirectional synaptic plasticity mechanism. We employed the answer to this question to arrive at a calcium-dependent plasticity rule (CDPR) for the *h* conductance such that firing rate homeostasis was maintained when *h*-channel plasticity accompanied synaptic plasticity. Finally employing calcium-dependent plasticity rules for synaptic strength and *h* conductance magnitude, we demonstrate that the synergy between these two plasticity rules accomplish much more than firing rate homeostasis. Specifically, we show that the co-occurrence of the two forms of plasticity enabled retention of synaptic weights within a useful dynamic range, introduced metaplasticity in the synaptic learning rule so that the positive feedback introduced by repeated synaptic potentiation was nullified, and facilitated reliable rate-based information transfer across the neuron when faced with positive feedback introduced by repeated synaptic potentiation.

## Results

### Plasticity in *h* Conductance Maintained Firing Rate Homeostasis after Bidirectional Synaptic Plasticity

**Figure 1:**
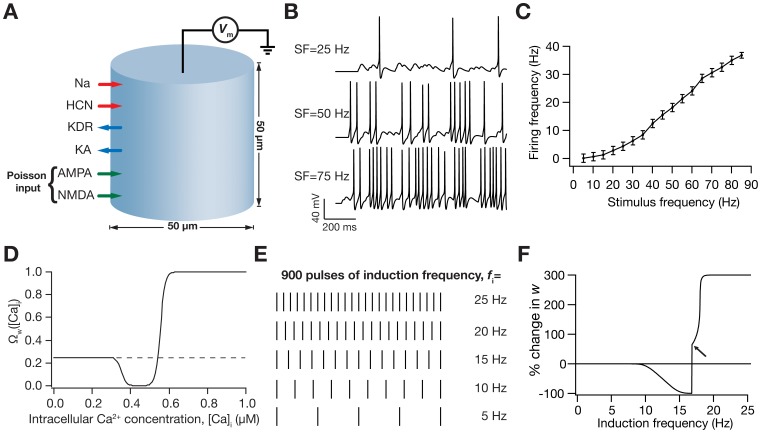
Illustration of the model and its basic properties. (**A**) Schematic of the single compartment model used in this study. The various ligand- and voltage-gated channels used in the model are depicted as arrows, and the transmembrane voltage Vm was recorded in response to Poisson-modulated excitatory synaptic inputs or to pulse current injections. (**B**) Voltage traces (for 1 s) depicting neuronal firing for Poisson-distributed synaptic stimulation at various stimulus frequencies (SF). (**C**) Plot showing firing frequency (FF) as a function of SF. Data represented as mean ± SEM for 100 trials of Poisson-distributed synaptic stimulation at each SF. (**D**) Functional form of the plasticity-regulating 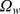 (Equation 14) plotted as a function of intracellular calcium levels. (**E**) Synaptic plasticity was induced by stimulating the colocalized NMDAR-AMPAR synapses with 900 pulses of various induction frequencies (*f*_i_) spanning a range of 0.5–25 Hz. Depicted are pulses for five different frequencies for a period of 1 s. As the number of pulses was set at 900 irrespective of the induction frequencies, induction of plasticity through a lower frequency pulse will run for a longer time compared to induction through a higher frequency pulse. (**F**) BCM-like synaptic weight (*w*) change induced through various induction frequencies. Arrow indicates the initiation of spikes during the induction protocol.

What changes in the *h* conductance are required for it to maintain firing rate homeostasis, when it is perturbed by bidirectional synaptic plasticity? What should be the relationship between HCN channel plasticity and synaptic plasticity for the former to counteract the perturbation in the input-output relationship that was imposed by the latter? To answer these questions, we employed a conductance-based model of a hippocampal pyramidal neuron with ion channel kinetics derived from experimental measurements and inserted a synapse made of colocalized AMPAR-NMDAR in the model [13]. The synaptic drive to the model neuron (Fig. 1A) was modeled as Poisson-distributed pre-synaptic action potentials arriving at various stimulus frequencies (SF), with the output defined by the neuron’s firing frequencies (FF). We presented the model with 100 trials of inputs at each SF and measured the FF (Fig. 1B) to construct the input-output relationship of the baseline (target) model (Fig. 1C). Then, we induced synaptic plasticity in the model synapse through a biophysical plasticity rule that was driven by Ca^2+^ influx through NMDARs [6], [13]. This yielded a BCM-like plasticity profile as a function of induction frequency, *f*_i_, when plasticity was induced through 900 synaptic stimuli (Fig. 1D–F) at the given *f*_i_ [6], [13].

**Figure 2:**
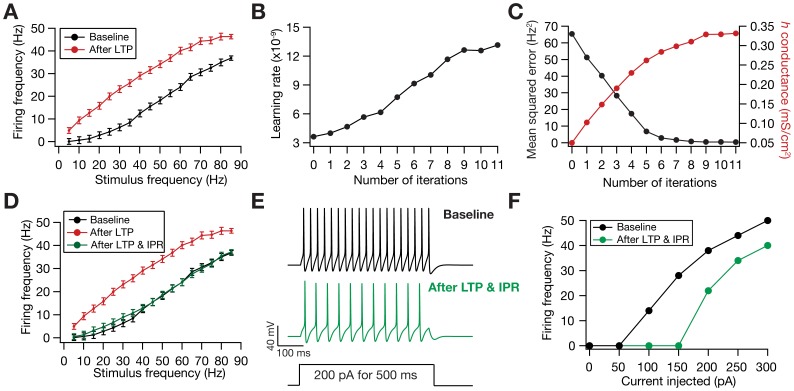
Plasticity in HCN channel conductance through the iterative plasticity rule (IPR) maintained firing rate homeostasis after LTP. (A) LTP induction (25 Hz/900 pulses) resulted in a leftward shift in the FF *vs*. SF plot. Black: Baseline; Red: after LTP induction. (B) Minimal learning rate (Equation 40) plotted as a function of number of IPR iterations. (C) Evolution of *h* conductance and the corresponding mean-squared error in the FF-SF plot depicted as functions of IPR iterations. (D) FF *vs*. SF plots under baseline condition (black), after LTP induction (red), and after LTP induction and IPR. (E) Traces showing neuronal firing for a non-synaptic pulse current injection of 200 pA, before (black) and after IPR (green). (F) Firing frequencies for various amplitudes of non-synaptic pulse current injections (of 500 ms) shown for cases before (black) and after IPR (green). Note that the induction of LTP alone does not alter neuronal response to non-synaptic pulse current injection. 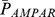 = 10 nm/s and baseline 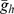 = 0.05 mS/cm^2^.

When long-term potentiation (LTP) was induced in the model synapse through 900 stimuli at 25 Hz (Fig. 1F), the FF-SF plot shifted towards the left (Fig. 2A) as a direct consequence of increased AMPAR conductance. This constitutes a perturbation in the FF of the neuron for given synaptic drive, and activity homeostasis requires that FF returned to its target levels for all SFs. Our goal was to quantitatively assess the validity of the postulate that changes in the *h* conductance were sufficient for such compensation. To do this, we employed an iterative plasticity rule (IPR) for *h*-conductance plasticity based on a gradient descent algorithm to minimize the mean-squared error (MSE) between the target FF and post-LTP FF for all considered SFs [28]. We derived the IPR through a parameterization of FF-SF curve as a sigmoid (with slope parameter *a*, and shift parameter *b*) and empirically arriving at the dependence of the sigmoidal parameters on the *h* conductance, 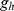 (see Models and Methods for the derivation of the IPR). Consequently, after the induction of LTP, we implemented the IPR over several iterations (*k*), involving an update of 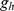 as in equation (24). The learning rate parameter 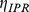 (Equation 24) was set to be lesser than a threshold, below which the IPR would stably converge (see Models and Methods for the derivation based on the Lyapunov stability criterion; Equation 40).

When we applied IPR on the *h* conductance of the model after the synapse underwent LTP (Fig. 2A), we found that the minimum learning rate (Equation 40) evolved as a function of activity (Fig. 2B) and *h*-conductance increased in the process of reducing the MSE (Fig. 2C). This reduction in MSE translated into maintenance of firing rate homeostasis, whereby FF at all SFs converged towards their target rates as an effect of plasticity in *h* conductance (Fig. 2D). Finally, whereas HCN channel plasticity through IPR was geared towards maintaining the input-output relationship with the synapses forming the input end, experimental results show a post-LTP reduction in neuronal intrinsic excitability assessed by direct, pulse-current injections to the neuron [25], [26]. Conforming to these experimental findings, our results also showed reduced excitability with LTP (Fig. 2E–F), implying a reduction in overall neuronal excitability that spans all synapses in the neuron.

**Figure 3:**
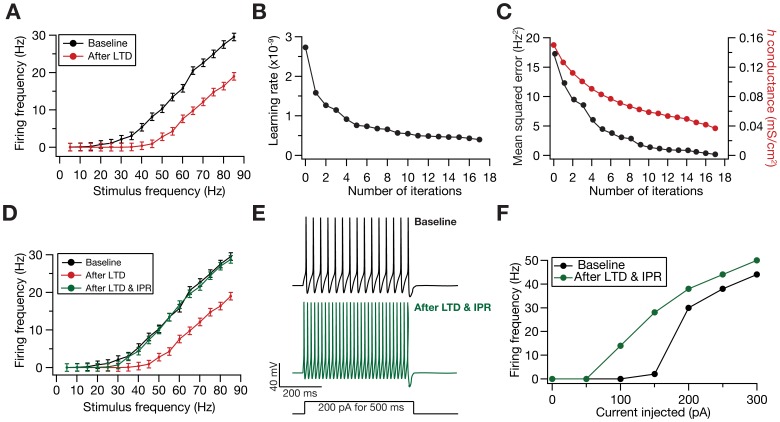
Plasticity in HCN channel conductance through the iterative plasticity rule (IPR) maintained firing rate homeostasis after LTD. (A) LTD induction (15 Hz/900 pulses) resulted in a leftward shift in the FF *vs*. SF plot. Black: Baseline; Red: after LTD induction. (B) Minimal learning rate (Equation 40) plotted as a function of number of IPR iterations. (C) Evolution of *h* conductance and the corresponding mean-squared error in the FF-SF plot depicted as functions of IPR iterations. (D) FF *vs*. SF plots under baseline condition (black), after LTD induction (red), and after LTD induction and IPR. (E) Traces showing neuronal firing for a non-synaptic pulse current injection of 200 pA, before (black) and after IPR (green). (F) Firing frequencies for various amplitudes of non-synaptic pulse current injections (of 500 ms) shown for cases before (black) and after IPR (green). Note that the induction of LTD alone does not alter neuronal response to non-synaptic pulse current injection. 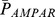 = 10 nm/s and baseline 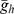 = 0.15 mS/cm^2^.

Our results above demonstrated that an increase in *h* conductance was sufficient to compensate for perturbations to the input-output relationship caused by LTP induction. However, a homeostatic role for HCN channels could be assigned only when such compensations were bidirectional. To assess this, we induced LTD through 900 synaptic stimuli at 15 Hz, and asked if changes in *h* conductance through IPR could compensate for the rightward shift to the FF-SF plot caused by LTD induction (Fig. 3A). We found that a reduction in *h* conductance was sufficient to compensate for this perturbation (Fig. 3B–C), thus accounting for bidirectionality of such plasticity and validating the postulate on a homeostatic role for HCN channels (Fig. 3D). Further, when we assessed firing frequency as a function of pulse-current injections, we found an increase in neuronal excitability with LTD (Fig. 3E–F), consistent with experimental findings [24]. We also performed sensitivity analyses across various baseline values for synaptic and HCN channel conductances, and found that the IPR retained homeostasis across a range of these baseline parameters (Fig. S1).

### HCN Channel Plasticity is Linearly Dependent on Synaptic Plasticity for Maintaining Firing Rate Homeostasis

**Figure 4:**
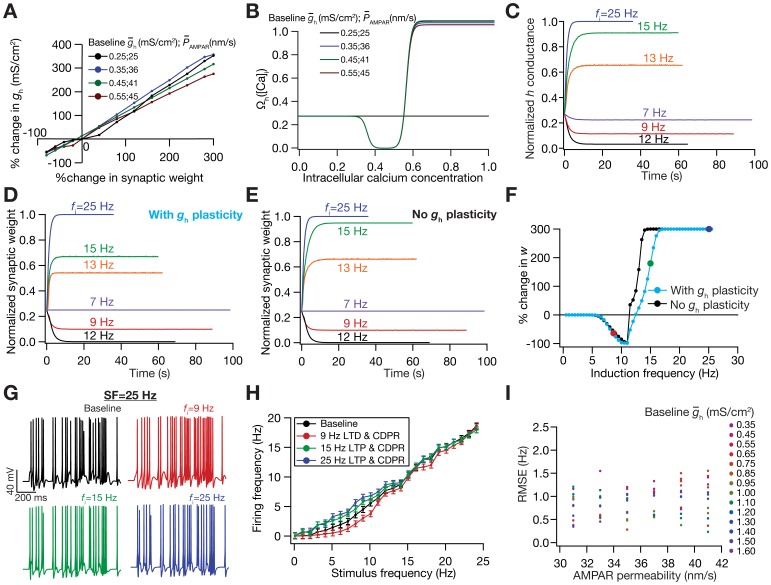
Calcium-dependence of *h*-conductance plasticity was deduced from the linear relationship between synaptic plasticity and HCN channel plasticity required for maintaining firing rate homeostasis. (A) Change in *h* conductance required to achieve homeostasis in the FF *vs*. SF plot was linearly related to the change in the synaptic weight for different induction frequencies (*f*_i_), for a range of values of 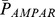 and baseline 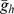. (B) Plot showing 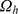 function (in Equation 41) as a function of [Ca^2+^]_i_. Note that the color codes for traces in (A) and (B) are the same. (C–D) Temporal evolution of normalized *h* conductance (C) and normalized synaptic weight, *w* (D), when the model was stimulated with 900 pulses of different values of *f*_i_. Plots depict the case where synaptic plasticity and CDPR were both induced in parallel. (E) Same as (D), but plots depict the case when only synaptic plasticity was induced. (F) BCM-like synaptic plasticity profiles, with (cyan) and without (black) CDPR. (G) Voltage traces depicting neuronal firing for a 25 Hz stimulus. Top left: baseline. The other three traces depict neuronal firing after parallel Ca^2+^-dependent induction of synaptic and HCN channel plasticity. Induction frequencies for top right: 8.5 Hz; Bottom left: 15 Hz; Bottom right: 19 Hz. Refer to color codes in (F). (H) FF *vs*. SF plots after induction of different magnitudes/direction of synaptic plasticity, when CDPR was induced in parallel, compared with baseline FF *vs*. SF (black). Note that the color codes representing different frequencies are the same from (C)–(H). (I) Sensitivity analysis of CDPR for 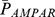 and baseline 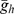, showing the root mean square error in firing rates between the baseline FF-SF plot and the one obtained after inducing LTP (25 Hz/900 pulses) in parallel with CDPR.

With these results providing quantitative evidences in support of the postulate for a homeostatic role for HCN channels, we next investigated the relationship between plasticity in the *h* conductance in maintaining activity homeostasis and the underlying synaptic plasticity that caused the perturbation to the activity. To do this, we induced synaptic plasticity using several values for *f*_i_ (Fig. 1E–F), and employed the IPR to assess the amount of HCN channel plasticity required for maintaining activity homeostasis for each value of *f*_i_. We found that the amount of HCN channel plasticity required for maintaining activity homeostasis was linearly related to the amount of synaptic plasticity that induced the perturbation (Fig. 4A). Such linear relationship was surprising because of all the nonlinearities that underlie the model under consideration, especially in terms of the FF-SF relationship (Fig. 1C) and the voltage-dependence of the HCN channel.

### A Calcium-dependent, *h*-conductance Update Mechanism that Accompanied Bidirectional Synaptic Plasticity Maintained Firing Rate Homeostasis

The analysis employing the IPR was geared towards the purpose of validating the postulate on a homeostatic role for the *h* conductance and towards understanding the relationship between synaptic and HCN channel plasticity. To do that, we had computed the change required in *h* conductance to maintain firing rate homeostasis *after* perturbing it through synaptic plasticity. However, experiments have demonstrated that synaptic plasticity and plasticity in measurements dependent on HCN channels evolve together as a function of time, with both forms of plasticity dependent on postsynaptic Ca^2+^ influx [24], [25], [26]. Taking these experimental results and the linear relationship between synaptic and *h*-conductance plasticity (Fig. 4A) together, we hypothesized that the dependence of plasticity in *h*-conductance on intracellular Ca^2+^ concentration ([Ca]_i_) would be analogous to the corresponding dependence of synaptic plasticity on [Ca]_i_. Specifically, we postulated that moderate levels of [Ca]_i_, higher than a certain depression threshold, would result in *h*-conductance depression and even higher levels of [Ca]_i_ would result in *h*-conductance potentiation.

Our results with the IPR provided us a quantitative foundation for testing this postulate. We employed the linear relationship between *h*-channel and synaptic plasticity (Fig. 4A) in conjunction with the calcium-dependent synaptic plasticity rule that we have employed [6], [13] to arrive at a calcium-dependent plasticity rule (CDPR) for *h*-conductance plasticity (see Models and Methods for the derivation of the CDPR). In deriving this rule, we noted that the slope of the linear relationship between *h*-conductance and synaptic plasticity was variable and depended on the values of baseline HCN channels conductance and values of permeability of the AMPAR. We accounted for this variability and derived a generalized rule that worked under various baseline values (see Models and Methods for more details). The CDPR that we used for the normalized *h* conductance *w*_h_ was as in equation (41). As a consequence of the differences in slope of the linear relationship across different baseline parameters (Fig. 4A), the 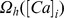 function displayed different levels of saturation for different baseline parameters (Fig. 4B).

We then tested if the CDPR was effective in terms of maintaining firing rate homeostasis when synaptic plasticity was induced through 900 pulses of various values of *f*_i_. To do this, we updated both the synaptic weight and the *h* conductance *simultaneously* through respective Ca^2+^-dependent mechanisms. Consistent with the linear relationship that was the basis for the CDPR, we found that *h* conductance evolved in a manner (Fig. 4C) similar to synaptic weight (Fig. 4D) across different values for *f*_i_. We noted that plasticity in *h* conductance concurrent with plasticity in synapses through the induction protocol would mean a metaplastic shift in the synaptic plasticity profile, given the ability of HCN channels to modulate Ca^2+^ influx [13]. As expected, implementing CDPR in parallel to synaptic plasticity induced a shift in the synaptic plasticity profile (Fig. 4E–F), in opposite directions for LTP and LTD owing to the direction of change in *h* conductance. Finally, given that CDPR was implemented in parallel with synaptic plasticity, we found that *h-*channel plasticity had compensated for the perturbation induced by synaptic plasticity, across the range of tested values of *f*_i_ and over a range of values for baseline conductances (Fig. 4G–I; Fig. S2). Finally, similar to our results with the IPR (Figs. 2 and 3), we also confirmed that CDPR induced an increase in intrinsic excitability with synaptic depression, and a reduction in intrinsic excitability with synaptic potentiation (Fig. S2). In summary, our results demonstrated that firing rate homeostasis was maintained by HCN channel plasticity when CDPR accompanied bidirectional synaptic plasticity.

### Synaptic Weights were Retained in a Useful Range as a Consequence of CDPR Accompanying Synaptic Plasticity

The CDPR for HCN channel plasticity was derived with a goal of retaining firing rate homeostasis in the face of bidirectional synaptic plasticity. Are there any other consequences of implementing firing rate homeostasis through HCN channel plasticity? We already noted that neuronal intrinsic excitability changed in a direction opposite to synaptic plasticity (Fig. S2), and that metaplasticity was induced when CDPR was implemented simultaneously along with synaptic plasticity (Fig. 4E–F). What are the consequences of this metaplasticity that accompanies synaptic plasticity? Could the shift in synaptic plasticity profile introduce stable synaptic learning, by retaining weights in a useful dynamic range?

**Figure 5:**
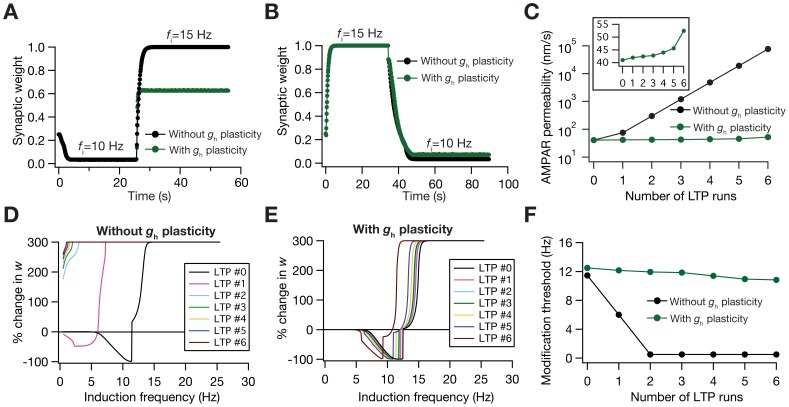
Calcium-dependent plasticity in HCN channels retains dynamic range of the synapses in the face of synaptic plasticity. (A) Experiment assessing the role of abrupt change in activity on synaptic weight dynamics, with initial induction frequency set at 10 Hz, and switched to 15 Hz after weight reached stable equilibrium with the 10 Hz stimulus. Traces show the cases where synaptic plasticity was accompanied (green) and not accompanied (black) by CDPR. (B) Same as (A), but with the initial and the switch frequencies reversed. (C) Impact of repeated LTP (15 Hz/900 pulses, each induction) on AMPAR permeability depicted as a function of the number of LTP inductions, plotted for cases where synaptic plasticity was accompanied (green) and not accompanied (black) by CDPR. Inset shows the same green plot on a linear scale. (D–E) Effect of repeated LTP on the BCM-like synaptic plasticity profile, plotted for cases where synaptic plasticity was not accompanied (D) and accompanied (E) by CDPR. (F) Modification threshold, calculated from traces shown in (D) and (E), plotted as a function of the number of LTP inductions for cases where synaptic plasticity was accompanied (green) and not accompanied (black) by CDPR. 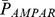 = 41 nm/s and baseline 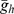 = 0.45 mS/cm^2^.

We addressed these questions through multiple approaches. First, we assessed the temporal evolution of synaptic plasticity when the SF was abruptly switched [17], with and without CDPR being implemented in parallel (Fig. 5A–B). Doing this, we found that synaptic weight values were closer to saturating limits when CDPR did not accompany synaptic plasticity. However, when CDPR accompanied synaptic plasticity, the synaptic weight was spared from reaching its saturating limit value (Fig. 5A), thus retaining its ability to change in response to further plasticity-inducing stimuli. It may be noted that amount of change with LTD was minimal (Fig. 5B), owing to design of the synaptic plasticity protocol (Fig. 1D–F). Second, we directly tested if the presence of CDPR and the consequent metaplasticity (Fig. 4E–F) can alleviate the instability caused by positive feedback loop induced by repeated induction of LTP. Specifically, it is known that synaptic potentiation increases AMPA receptor density, which results in increased Ca^2+^ influx, thus causing more potentiation for the same LTP-inducing stimulus that arrives after the first potentiation [6], [13], [17], thus leading to a saturation in synaptic weight. When CDPR was not accompanying LTP-inducing stimuli, repeated presentation of the stimulus led to a runaway increase in synaptic weight. However, when CDPR accompanied the consecutive LTP-inducing stimuli, the synaptic weight did not undergo runaway increase, owing to the metaplastic effects of CDPR discussed above (Fig. 5C).

How do the metaplastic effects manifest themselves when presented with repeated LTP-inducing stimulus? To answer this, we repeatedly induced LTP and generated the synaptic plasticity profile after each LTP-inducing stimulus. As increase in AMPAR permeability induces a leftward shift in the plasticity profile [6], [13], in the absence of synergistic HCN channel plasticity, repeated induction of LTP and the associated runaway increase in AMPAR permeability (Fig. 5C) shifted the plasticity profile to an extent where all values of *f*_i_ led only to LTP (Fig. 5D). This hampers the bidirectional nature of synaptic plasticity, thus rendering the plasticity profile incapable of stable learning. However, when CDPR accompanied LTP, the leftward shift introduced by increased AMPAR (which was not as high as the case without CDPR; Fig. 5C) was largely compensated by the rightward shift introduced by increased *h*-conductance [13]. This led to a homeostasis in plasticity profile, where the bidirectional nature of the BCM-like plasticity profile was conserved and the profile remained largely invariant through the successive LTP inductions (Fig. 5E–F). Thus, HCN channel plasticity, accompanying synaptic plasticity through CDPR, apart from fulfilling its designed purpose of maintaining firing rate homeostasis, also retained synaptic weights within a useful range and maintained plasticity homeostasis to conserve the stable bidirectional nature of the plasticity profile.

### Information Transfer Across the Neuron was Robust When CDPR Accompanied Synaptic Plasticity

**Figure 6:**
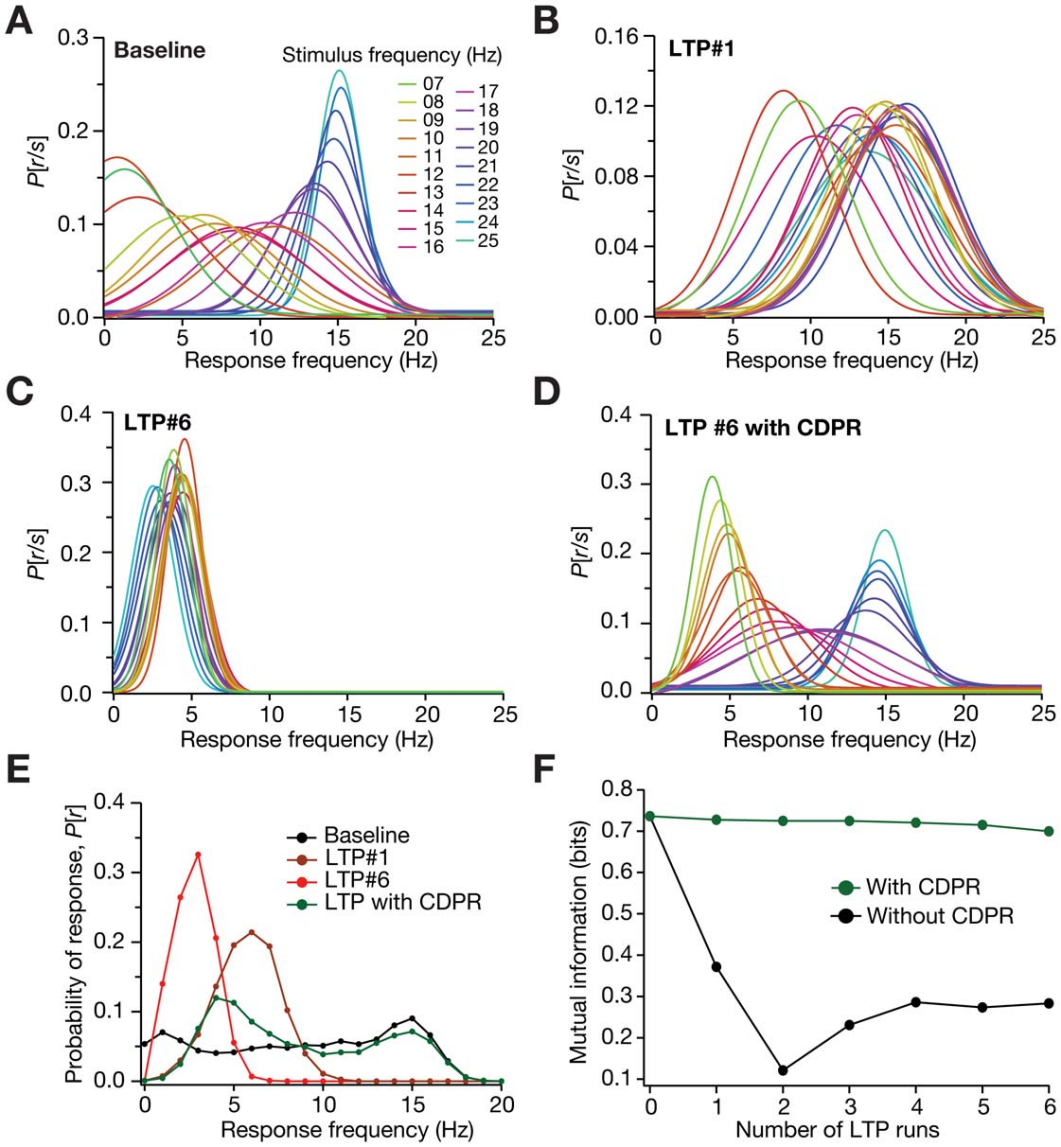
Information transfer across the neuron was more robust when synaptic plasticity was accompanied by HCN channel plasticity. (A) Probability distribution of response firing frequency, given SF, *P* [*r*|*s*], with baseline parameters. (B–C) *P* [*r*|*s*] after induction of LTP (B; 15 Hz/900 pulses), and after six consecutive LTP inductions (C; 15 Hz/900 pulses, each induction). Synaptic plasticity was not accompanied by CDPR for both cases. (D) *P* [*r*|*s*] after six successive LTP inductions, with CDPR induced in parallel with synaptic plasticity. For *P* [*r*|*s*] depicted in panels (A–D), the individual normal distributions were constructed from the first and second order statistics of the FFs, across trials, for a given SF. (E) Probability distribution for different response frequencies, *P* [*r*], plotted for baseline condition (black), after a single run of LTP induction (brown), and after six consecutive LTP inductions accompanied (green) and not accompanied (red) by CDPR. (F) Mutual information plotted as a function of number of successive LTP runs, under cases where CDPR accompanied (green) or did not accompany (black) synaptic plasticity. 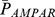 = 41 nm/s and baseline 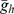 = 0.45 mS/cm^2^.

What are the consequences of HCN channel plasticity and the consequent stability to neural coding? To answer this, we considered a rate-coding scheme, where the model neuron encoded incoming information, arriving as stimulus firing rate, by modulating its own output firing rate. Mutual information was then calculated as an estimate of the accuracy with which the stimulus can be encoded by the neuron’s response. The input presented to such a system was through the stimulation of the model synapse with Poisson-distributed spike trains at different SFs, and mutual information was computed by considering the neuronal output frequency as the response. Under such a rate-coding scheme, we presented the synapse with repeated LTP-inducing stimuli, and asked how mutual information evolved with every run of LTP. In the absence of HCN channel plasticity, given the runaway excitation that accompanied repeated LTP (Fig. 5C) and the consequent shift in the FF-SF plot, we found that the response frequency was rendered non-discriminatory (across SFs) with increase in LTP runs (Fig. 6A–C). This, accompanied by a reduction in the dynamic range of the response (Fig. 6E) meant that information transfer across the neuron deteriorated with increase in LTP runs (Fig. 6F). However, when CDPR accompanied LTP, the discriminatory capability (Fig. 6D) and the dynamic range of the response frequency (Fig. 6E) were conserved, thus enabling robust information transfer across the neuron in the face of repeated LTP. Finally, we performed sensitivity analyses to ascertain if our results on the ability of CDPR to avert runaway excitation (Fig. 5), and to retain robustness of information transfer (Fig. 6) in the face of repeated potentiation were robust to baseline parametric variation. Our results (Fig. S3) suggest that these results held for a range of baseline parameters, thus establishing the robustness of CDPR.

## Discussion

**Figure 7:**
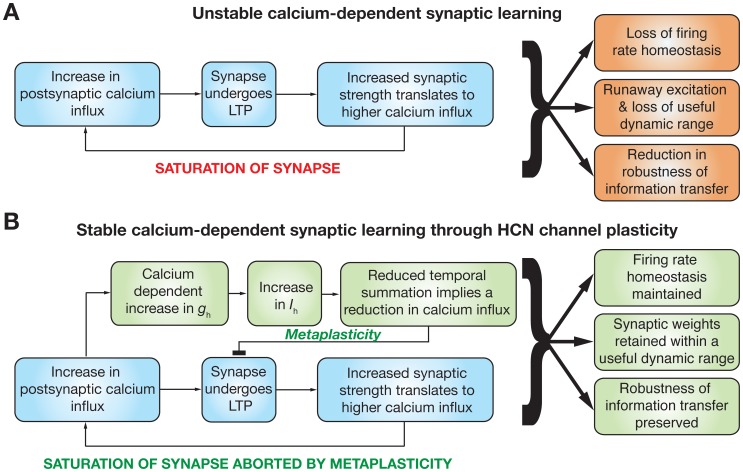
Summary diagram illustrating the physiological implications for the calcium-dependent plasticity rule governing changes in the *h* current, and its interactions with calcium-dependent synaptic plasticity. (A) In the absence of a homeostatic mechanism, the calcium-dependent synaptic plasticity rule acts as a positive feedback loop. Increase in synaptic weight through LTP leads to further enhancement of synaptic strength given the direct dependence of calcium influx on synaptic strength, and the dependence of synaptic plasticity on the calcium influx. Thus repeated potentiation leads to saturation of synaptic strength. Whereas the diagram depicts the case for LTP, a similar diagram would follow for LTD as well, and synapses would die in the case of repeated depression. This positive feedback loop would thus lead to a loss of firing rate homeostasis (Figs. 2 and 3), saturation/death of synapses (during repeated potentiation/depression respectively) ensuring that synapses do not retain a useful dynamic range (Fig. 5), and a loss in the robustness of information transfer across the neuron under a rate-coding schema (Fig. 6). (B) Under the scenario where the HCN channel conductance (*g*_h_) was updated using CDPR, and was updated in parallel to changes in synaptic strength, change in *h* current (*I*_h_) alters excitability and temporal summation thereby modulating calcium influx into neurons. This change in calcium influx introduces a metaplastic shift to the synaptic plasticity profile [13] and forms a negative feedback mechanism that counters the positive feedback associated with the calcium-dependent synaptic plasticity in (A). Thus, HCN channel plasticity through CDPR acts as a homeostatic feedback mechanism ensuring that there is retention of firing rate homeostasis (Figs. 2, 3, and 4), of synapses in a useful dynamic range (Fig. 5), and of the robustness of information transfer across the neuron (Fig. 6).

In this study, we have quantitatively established a homeostatic and stability-promoting role for HCN channel plasticity, which has experimentally been demonstrated to accompany synaptic plasticity. In establishing this, we derived biophysically rooted plasticity rules for the HCN channel, through which we present our hypothesis that the direction and magnitude of HCN channel plasticity are dependent on the levels of postsynaptic Ca^2+^ influx. We also demonstrated that HCN channel plasticity accompanying synaptic plasticity could play multiple parallel roles, in terms of retaining firing rate homeostasis, altering intrinsic excitability, retaining synaptic weights in a useful dynamic range by inducing metaplasticity, and enabling robust information transfer across a neuron under a rate coding model. These, accompanied by other well-known physiological roles for HCN channels [29], establishes the HCN channel as a powerful regulator of neural coding, learning and homeostasis (Fig. 7).

### Physiological Relevance of the Calcium-dependent Update Rule of HCN Channels

Our Ca^2+^-dependent plasticity rule for HCN channels states that beyond a certain threshold, moderate levels of Ca^2+^ influx induce *h*-conductance depression, whereas higher levels of Ca^2+^ influx induce *h*-conductance potentiation. Although this rule was derived purely from the perspective of maintaining firing rate homeostasis, there are multiple lines of experimental evidence to support this plasticity rule. Prominent among these is the existence of bidirectional plasticity in the *h* current, wherein a low-frequency pairing protocol that induces LTD in the hippocampus was accompanied by a reduction in the *h* current [24], whereas a high-frequency pairing protocol that induces LTP was accompanied by an increase in the *h* current [25], [26]. In another line of evidence, when plasticity in *h* current was assessed as a function of the magnitude of LTP induced, it was found that weaker and stronger LTP were accompanied by a reduction and an increase in the *h* current, respectively [30]. These, accompanied by the large body of literature on Ca^2+^-dependent synaptic plasticity and how different levels of Ca^2+^ modulate synaptic plasticity in the hippocampus [4], [6], provide considerable evidence to our postulate on HCN channel plasticity as a function of Ca^2+^ levels. Further, based on the design of the CDPR, the temporal evolution of both the homeostatic plasticity driven by HCN channels and the synaptic plasticity are concurrent. Whereas this is contrary to the dogma that homeostatic plasticity is slower than mnemonic plasticity, there is evidence in the literature where both such forms of plasticity can evolve concurrently [15]. More importantly, synaptic efficacy and measurements that are sensitive to HCN channels evolve concurrently in cases where both have been shown to accompany each other [24]–[26], thus providing experimental evidences to our conclusions on concurrent and dynamic gain control accompanying synaptic plasticity.

### Implications for Calcium-dependent Plasticity in HCN Channels

The coexistence of synaptic and intrinsic plasticity mechanisms within a neuron is now well established, with several postulates on how these two mechanisms could synergistically interact under several physiological and pathophysiological conditions. One viewpoint has been that intrinsic plasticity could be broadly classified into two categories: mnemonic, where plasticity in intrinsic properties participate in the encoding process; and homeostatic, where its role is in promoting stability during the learning process [10]. Based on experimental evidences and prior hypotheses, we postulated a homeostatic role for HCN channel plasticity to quantitatively validate the postulate and assessed the other consequences of such plasticity. In doing so, our analysis of HCN plasticity was limited to changes in excitability and activity homeostasis, to stable synaptic learning and to information transfer under a rate-coding schema (Fig. 7). Future studies could expand such analysis to the impact of HCN plasticity on other physiological aspects governed by these channels, including their regulation of resting membrane potential [31], [32], [33], [34], of theta-frequency resonance and phase modulation [26], [35], [36], and of post inhibitory rebound [37].

Although HCN channel plasticity is relatively well studied, it is just one among the several VGICs that have been demonstrated to change during learning protocols or under pathological conditions. Future studies could focus on deriving rules for these other channels, and assess the synergy of plasticity across different channels, including synaptic receptors, exploring their homeostatic/mnemonic roles with implications to learning theory and to neural encoding [10], [16], [28], [38], [39]. These studies could also account for possible local plasticity in various ion channels, which would require morphologically equivalent models, as opposed to global plasticity [24], [26] in HCN channels studied here. As recent literature has made it abundantly clear that the same physiological properties could be obtained through several nonunique combinations of underlying VGICs [31], [33], [34], [40]–[42], it is also important to understand how different VGICs and receptors interact with each other, and how their plasticity rules are linked to each other towards achieving the twin goals of information encoding and homeostasis [43]. Finally, considering parallels from the synaptic plasticity literature [3], such studies should also endeavor to go beyond a simple Ca^2+^-dependent model for plasticity, and account for Ca^2+^ kinetics, downstream signaling pathways, microdomains associated with these signaling pathways, protein synthesis and trafficking to understand plasticity in various VGICs and their synergies with synaptic plasticity. This is extremely critical because plasticity in different channels and receptors occur in unison with the same plasticity inducing protocols [26], [35], [44], [45], because they are linked to each other by specific signaling molecules, their kinetics and their subcellular locations [3], [46].

### Emergent Features of the Calcium-dependent Plasticity Rule for HCN Channels

Although our calcium-dependent plasticity rule was designed for achieving firing rate homeostasis, features emergent from the interactions between HCN plasticity and synaptic plasticity implied several additional functional implications for the synergy between the two forms of plasticity (Fig. 7). First, when HCN plasticity accompanied synaptic plasticity, the saturation of synapses that followed repeated synaptic plasticity was aborted, ensuring that synaptic strengths remained in a useful dynamic range (Fig. 5). In synaptic learning systems, it is critical that neurons and their networks retain their ability to learn, and this in turn depends on the learning process retaining synaptic weights within a useful dynamic range. Whereas our results indicate that plasticity in VGICs could act as a mechanism that enforces this in single neuron models through metaplasticity (Fig. 5), it would be important to understand the implications for HCN plasticity in learning networks and their homeostasis. Although network homeostasis has been analyzed through synaptically driven mechanisms [1], [7], [14], [17]–[21], roles for VGICs and their plasticity in network homeostasis need exploration through computational models. Thus, future studies could explore the role of HCN plasticity through CDPR in network homeostasis employing biophysically constrained models for neurons and for synaptic plasticity. In conjunction with experimentally constrained activity patterns, this would provide insights into the roles of HCN channels and their plasticity in regulating synaptic homeostasis in networks under physiological conditions as well as pathophysiological conditions such as depression and epilepsy [47]–[50].

The second feature that emerges from the interactions between the two forms of plasticity is that HCN plasticity enables neurons to preserve the robustness of information transfer in the face of repeated synaptic plasticity (Fig. 6). The ability of neurons to encode information, and their ability to maximize mutual information has been an active area for investigation across various neuroscience disciplines [51]–[59]. Although the role of VGICs in assessing information maximization has been suggested and analyzed through generic intrinsic plasticity mechanisms [53], [60], experimentally driven rules for specific VGICs have not been employed to analyze information maximization in underlying neuronal systems. Our results suggest that plasticity in HCN channels in conjunction with synaptic plasticity preserves the robustness of such information transfer in the face of repeated synaptic plasticity (Fig. 6). These results present several avenues for future explorations linking various forms of plasticity in specific VGICs to neural coding in general and information maximization in particular. Specifically, future studies could focus on assessing the relationship between learning rules that maximize mutual information and those that maintain firing rate homeostasis, with reference to the VGIC that is being studied. For instance, whereas our update rule for HCN channels was designed to maintain firing rate homeostasis, we also observed that mutual information is preserved in the face of repeated synaptic plasticity. Broadly, these results suggest that homeostasis in input-output relationship is essential to maintain robustness in the transfer of information across the neuron. It would be of interest to test if the converse, that retention of robust information transfer across neurons would establish homeostasis in the input-output relationship, is also true so as to establish a quantitative tight relationship between neural coding and homeostasis. Specifically, future studies could focus on the question on whether an update rule for HCN channels derived specifically to maximize mutual information would also maintain firing rate, and ask if a generalized relationship exists between rules that maximize mutual information and those that maintain firing rate [53], [60]?

From the standpoint of neural coding, analyses of information maximization in neural systems have led to a class of algorithms that perform independent component analysis, and independent components that emerge from such algorithms have been linked to efficient encoding in neural systems [52], [54], [57], [61], [62], [63], [64]. In this context, although algorithms have been proposed for independent component analysis through synaptic and intrinsic plasticity mechanisms [54], [57], [65], none of them consider experimentally constrained plasticity rules for specific VGICs and their interactions with synaptic plasticity. Therefore, future explorations relating information maximization and VGIC plasticity could answer questions on whether experimentally constrained plasticity rules for specific VGICs in conjunction with synaptic plasticity could serve as substrates for information maximization and efficient coding of information.

In summary, in this study, we derived experimentally grounded plasticity rules for one of the most well studied VGIC, and demonstrated that the synergy between synaptic and VGIC plasticity retains stability in the synaptic learning system, and enhances the robustness of information transfer across the neuron. We also provided specific quantitative relationships between plasticity in this VGIC and the influx of calcium, for such stability to be maintained, and presented experimental evidence in support of the specific quantitative relationship that we had derived. Our study establishes a broad framework for the coexistence of synaptic and VGIC plasticity, and emphasizes the need for considering all forms of plasticity in a holistic manner in assessing neural coding, learning theory, homeostasis, and the pathophysiology of neurological disorders [10], [43], [50], [66]. Such analyses should also dissect the contributions of different forms of plasticity to specific physiological roles, by deriving specific rules for plasticity in each of these components and understanding the interactions between these components and their update rules.

## Models and Methods

A single compartmental, conductance-based neuronal model of length 50 µm and diameter 50 µm was used for all simulations. The reasons behind the choice of a single compartmental model for our simulations were two fold. First, given the computational complexity associated with the plasticity paradigms (detailed below), incorporating these into a morphological realistic model would constitute an enormous increase in computational cost given the increase in the number of neuronal compartments. Second, bidirectional plasticity in the *h* current has been demonstrated to be spatially widespread, encompassing synapses that have not been subjected to plasticity [24], [25], [26]. This global nature of *h*-current plasticity has also been shown to change measurements sensitive to *h* current by an equal percentage across the stretch of the apical trunk [26]. Therefore, we reasoned that implementing a morphologically realistic neuron and introducing uniform percentage of change in *h* conductances distributed throughout the morphology was conceptually equivalent to employing a single compartment model with an *h* conductance that was updated according to the plasticity rule. Therefore, it was the global nature of the experimentally observed *h* current plasticity that provided us the avenue to employ a single-compartmental model and use it as an abstraction to arrive at the plasticity rules that we present below. In employing this model for our simulations, we constrained parameters and kinetics of constitutive components with experimental measurements from CA1 pyramidal neurons, the details of which are provided below.

The passive parameters associated with the model were: *R_m_* = 28 kΩ cm^2^ and *C*_m_ = 1 µF/cm^2^. Fast Na^+^, delayed rectifier K^+^ (KDR), *A*-type K^+^ (KA) and HCN channels were introduced, with kinetics adopted from experimental measurements of these channels in hippocampal pyramidal neurons [13], [67]–[71]. Default values of maximum conductance densities (in mS/cm^2^) were set to qualitatively match (see Fig. 2F) the firing frequency *vs*. current (*f*-*I*) curves of hippocampal pyramidal neurons [26], 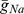 = 42, 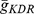 = 5, 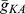 = 1, 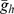 = 0.35. Reversal potentials for the *h*, Na^+^ and K^+^ channels, respectively were (in mV), *E*_h_ = –30, *E*_K_ = –90, *E*_Na_ = 55. All simulations were performed at –65 mV in the NEURON simulation environment [72], with an integration time constant of 25 µs.

### Synapse Model

A synapse was modeled as a co-localized combination of NMDA and AMPA receptor currents. A default value of NMDAR:AMPAR ratio was set at 1.5 and synaptic plasticity was induced in the model synapse by presenting 900 pulses at a various induction frequencies [6], [13], [73], [74]. The current through the NMDA receptor, as a function of voltage and time, was dependent on three ions: sodium, potassium and calcium. Consequently, as per the Goldman-Hodgkin-Katz convention [13], [75]–[78]:
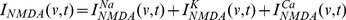
(1)where,



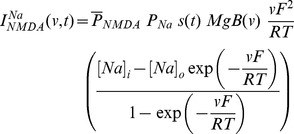
(2)

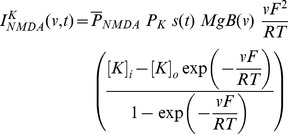
(3)

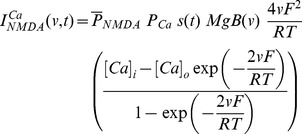
(4)where 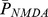 is the maximum permeability of the NMDA receptor. The relative permeability ratios 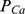 = 10.6, 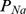 = 1, 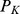 = 1, owing to the permeability of the NMDA receptor to different ions being 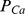: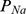: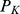 = 10.6∶1∶1. Default values of concentrations were (in mM): 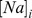 = 18, 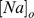 = 140, 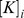 = 140, 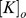 = 5, 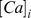 = 100 × 10^−6^, 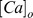 = 2. The concentration for sodium was set such that equilibrium potential was at +55 mV and that for potassium was at –90 mV. Evolution of intracellular calcium with NMDA-dependent calcium current 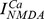 and its buffering was modeled as in [13], [79]:
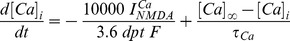
(5)where 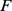
is Faraday’s constant, 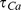 = 30 ms is the calcium decay constant, 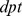 = 0.1 µm is the depth of the shell, and 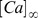 = 100 nM is the steady state value of 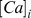. 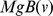
governs the magnesium dependence of the NMDA current, given as [80]:
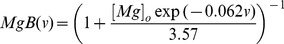
(6)with the default value of 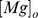
set at 2 mM. 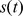
governs the kinetics of the NMDA current, and is given as:
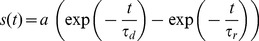
(7)where 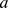 is a normalization constant, making sure that 0≤ 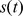
≤1, 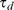 is the decay time constant, 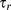 is rise time, with 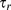 = 5 ms, and 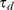 = 50 ms [13], [81].

Current through the AMPA receptor was modeled as the sum of currents carried by these sodium and potassium ions:
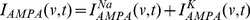
(8)where,



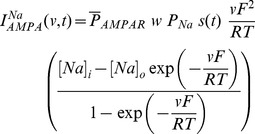
(9)

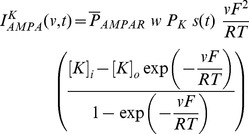
(10)where 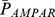 is the maximum permeability of the AMPA receptor, whose default value was set at 10 nm/s. The relative permeability ratios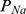 and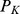 were equal and set to 1 [81]. 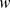
is the weight parameter that undergoes activity-dependent plasticity (see below). 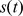
was the same as that for the NMDA receptor, but with 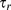 = 2 ms and 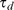 = 10 ms [13], [81].

### Induction of Synaptic Plasticity

Synaptic weight parameter *w* (see Equations 9 and 10) was updated as a function of intracellular calcium, following the calcium control hypothesis [4], [6], [13], [82]. Specifically,
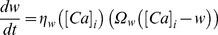
(11)where, 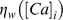 is the calcium dependent learning rate, inversely related to the learning time constant 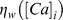:



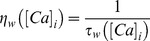
(12)

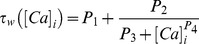
(13)with 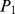 = 1s, *P*_2_ = 0.1s, 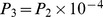, 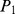 = 3. 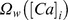 had the following form:
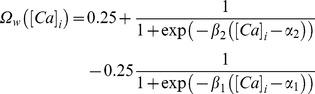
(14)with 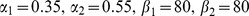. In all of the above weight update equations, for compatibility, 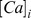
is set as 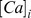
–100 nM. Unless otherwise stated, the default initial value of 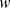, 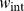, was set at 0.25.

Using this framework, the direction and strength of plasticity were analyzed by presenting stimuli made up of 900 pulses at various induction frequencies (*f*_i_ spanning 0.5–25 Hz), an experimentally well-established plasticity protocol [73], [74]. The evolution of weights given by equation (11) was monitored and the final weight at the end of the induction protocol was noted down for each frequency. The percentage difference between this final weight and the initial weight (0.25) was plotted against the induction frequency of the stimulus pulses to obtain the synaptic plasticity profile as a function of induction frequency [6], [13]. Figure 1F provides an example of such a plasticity profile generated with our model. For LTP saturation experiments (Figs. 5 and 6), unless otherwise stated, we induced LTP in every trial through a 15 Hz/900 pulses protocol. We did not assess the implications of LTD saturation given that LTD-induced change in synaptic weight was minimal (*e.g.,* Fig. 1F), owing to the definition of the 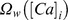 function (Equation 14).

### Derivation of Iterative Plasticity Rule (IPR) for g_h_ Update

In our model, we employed neuronal firing frequency (FF) as the output variable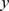, with the stimulus frequency (SF) constituting the input variable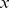. When input spike trains of *n* different SFs 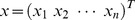 were fed to the synapse, our model postsynaptic neuron responded with corresponding FFs 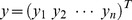, when 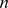 different SFs were employed in generating the FF-SF plot (Fig. 1B–C). In order to sustain firing rate homeostasis in the face of synaptic plasticity, this FF-SF plot ought to remain constant across different values of synaptic weights. To implement this, we defined 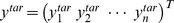 as the target firing frequency for the corresponding stimulus frequencies, and defined firing rate homeostasis as the ability of the model neuron to this target firing frequency across stimulus frequencies. Note that this target firing frequency is a function of the active and passive properties of the neuron, and the properties of the synaptic receptors, and would vary if any of the baseline parameters (*e.g.,* baseline 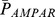 and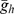) were altered. Now, if synaptic plasticity were induced and the FF-SF plot (**y**) changed as a consequence of this altered synaptic weight (*e.g.*, Fig. 2A), then, to maintain homeostasis, we need to minimize the mean square error (MSE), 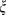, between 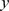
and 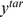:
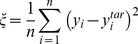
(15)


To minimize this function using the standard gradient descent algorithm, we needed a differentiable, parameterized form for the FF-SF curve [28]. Based on the structure of the FF-SF curve (Fig. 1C), we employed a sigmoid as a functional form to represent this input-output relationship. Specifically, the output firing frequency 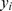 as a function of the input stimulus frequency 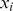 was written as a sigmoidal function:
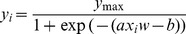
(16)where 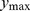 was the maximum firing frequency, 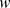 was the weight of the synapse under consideration, 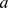 quantified the slope of the linear part of this sigmoid and 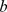 represented the offset in the curve. Under such parameterization, we chose slope 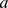 as the parameter over which 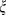 was minimized, because we empirically observed that the induction of synaptic plasticity significantly altered the slope in comparison to the offset of this function. In what follows, we derive the update equations for this parameter such that 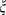 was minimized. First, differentiating (15) with respect to 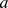:



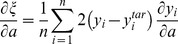
(17)Evaluating 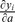 after plugging in the functional form for 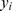 from (16), we have:
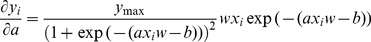
(18)


From (16), this reduces to:
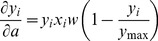
(19)


Rewriting (17),
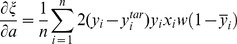
(20)where



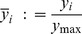
(21)To follow a gradient descent algorithm to minimize 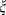 with respect to 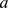, at each iteration, 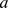 had to be updated such that we proceeded along the negative of this gradient direction. To be specific, at each iteration 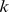, we implemented the following plasticity rule:
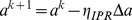
(22)where 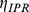 was the learning rate parameter for updating 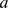, and 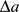 was as in (20):
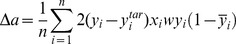
(23)Whereas the derivation thus far has focused on minimizing 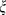 with reference to the slope parameter 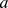, our goal was to minimize 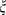, and thus achieve homeostasis, by changing the maximal h conductance 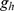. As we already had the update equations for 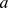, we needed the relationship between 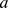 and 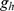 to derive the update equation for 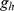. We estimated this relationship empirically by obtaining FF-SF profiles for various different values of 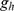, and parameterized these profiles as in (16) to obtain the corresponding values of 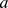. Upon doing this, we found that changes in 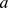 and 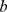 were linearly related to 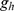 with different slopes 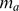 and 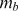 respectively. We employed this linear relationship to update 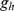 at each iteration 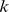 as follows:
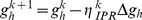
(24)where 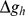 was given by:



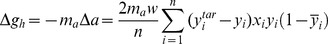
(25)As changes in 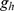 altered the FF–SF curve, this gradient descent approach enabled the minimization of 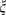, thus maintaining firing rate homeostasis after it was perturbed by the induction of synaptic plasticity. Although we updated HCN channel conductance to alter the FF–SF curve, and derived our optimization procedure for 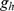, it should be noted that plasticity could be implemented through changes in the half-maximal activation voltage of the *h* conductance [13], [83]. As the goal was to alter the *h* current, we used conductance as a means to do this, although the procedure outlined above is generalizable to the half-maximal activation voltage of the *h* conductance.

### Derivation of Learning Rate Based on Lyapunov Stability Criterion

Under what constraints will the algorithm derived in the previous section converge? Is there a limit on the learning rate parameter 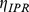 such that this algorithm stably converges? To answer these questions, we sought to derive constraints on the trajectory of our error function (Equation 15) to achieve stability of convergence in our dynamical update system. To do this, we considered the mean squared error function (Equation 15) as the Lyapunov function [84], [85] because the trajectory of this error with the adaptation in 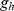 would determine the convergence of the iterative algorithm. Under such a framework, 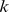, the iteration number during the gradient descent optimization procedure (above), would constitute the state of the system, and 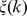 be the dynamics of the system, which would represent the energy function of the system, 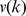.
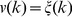
(26)where,
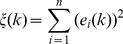
(27)and
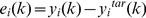
(28)where 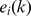 is the error function of the system.

Lyapunov theory states that if there is a function 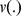 such that 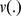 is positive definite and the gradient of the function, 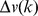, satisfied the criterion 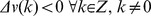 then, the system is globally asymptotically stable over the entire state space [84], [85]. Based on our choice of 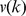 as the error function:
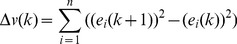
(29)Writing 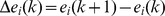, we obtained:
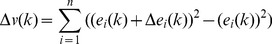
(30)which was rewritten as:



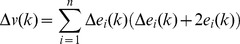
(31)As the gradient descent algorithm derived for 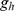 plasticity in (25) was based on minimizing the error between 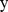 and 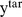, the change in error 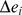 can be calculated from the change in 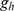 by writing:
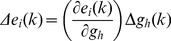
(32)



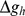
 was obtained from the gradient descent algorithm (above):
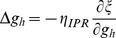
(33)


For a given SF 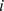 and given equation (27), we write.
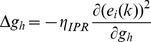
(34)


Substituting the value of 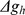 in (32),
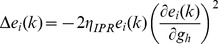
(35)


Substituting 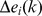 in (31),
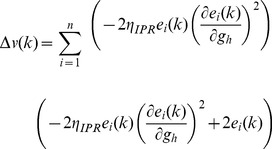
(36)


Simplifying this, we got,
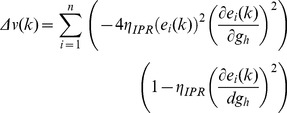
(37)

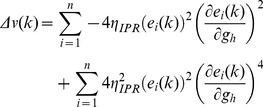
(38)


To satisfy the Lyapunov stability criterion, this gradient, 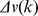, of the Lyapunov function has to be negative, which implies:
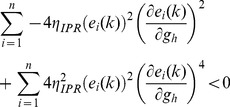
(39)


From this, we derived the constraint for the system to be globally asymptotically stable and ensuring convergence in terms of the learning rate parameter 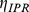 as:
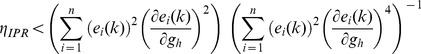
(40)



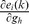
 was computed using the definition of 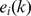 in (28), and in a manner similar to arriving at (19), and noting that changes in parameters 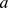 and 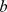 in (16) were linearly related to 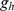 with different slopes 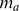 and 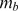
respectively.

Thus, the Lyapunov stability criterion would be satisfied, ensuring convergence of IPR, if the learning rate parameter satisfied the inequality provided in (40). To ensure this, in implementing the IPR, we always set 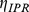 lesser than this value.

### Derivation of Calcium Dependent Plasticity Rule (CDPR)

The linear interaction between intrinsic and synaptic plasticity forms the basis for the derivation of the calcium dependent 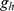 plasticity rule. The parameter 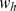 in (41) is similar to the calcium dependent synaptic weight update given in (11), this relation is implicated from the linear interaction between intrinsic and synaptic plasticity.
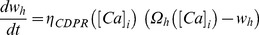
(41)where, 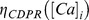 is the calcium dependent learning rate, inversely related to the learning time constant 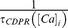:



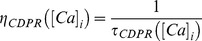
(42)

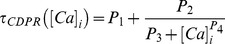
(43)with 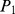 = 1 s, 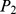 = 0.1 s, 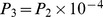, 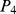 = 3. 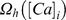 had the following form (Fig. 4B):
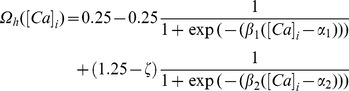
(44)with 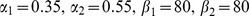. In all of the above weight update equations, for compatibility, 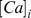 is set as 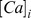
–100 nM. Unless otherwise stated, the default initial value of 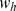, 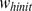, was set at 0.25. 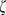 is the normalized h conductance as given as (Fig. S1E):
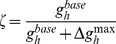
(45)where 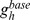 was the baseline h conductance, and 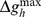 was the change in h conductance required for retaining homeostasis after the induction of maximal LTP (300%).

The rationale behind using 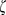 was as follows. If the relationship between the change in synaptic weight, 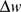, and the change required in *h*-conductance, 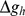, to maintain firing rate homeostasis after such synaptic plasticity were linear with a slope of 1, then 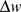 would be exactly equal to 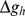 without any requirement for a offset parameter. However, from our results from analyzing the sensitivities of the IPR (Fig. 4A), it was evident that the slope was not always 1, and varied as a function of 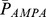 and 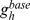, even though relationship between 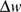 and 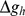 was linear across a range of parameters (Fig. 4A). Therefore, if 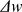 were 300%, then 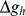 required for maintaining homeostasis would not be 300%. In order to account for such non-unity slopes for the linear relationship shown in Figure 4A, and thereby generalize our plasticity rule for a range of underlying parameters, we introduced this offset correction parameter 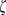. To arrive at 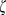 for any given parametric combination, we induced LTP (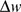 = 300%) with a 25 Hz/900 pulse stimulus and asked what %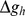 was required to retain firing rate homeostasis after this, using Figure 4A, and assigned that as 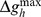. We then computed 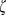 as in (45), using this 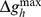 and the baseline *h*-conductance value 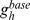. If the linear relationship between 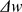 and 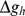 had unit slope, then 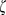, as defined in (45) would be 0.25 (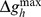 would be 300% of 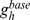), and 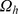 (Equation 44) would exactly be equal to 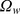 (Equation 11) thus letting 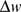 = 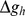. However, if the slope were not unity, this value would be away from 0.25 (Fig. S1E), and 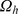 should be updated accordingly. As 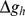 *vs* 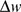 was a linear relationship, and we had two points on this straight line ((0,0) and (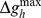, 300%)), we adjusted 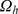 appropriately employing the linear relationship (Equation (44)–(45)).

Furthermore, as we wanted to retain 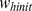, the initial value of 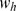, at 0.25 given the steady state conditions associated with this dynamical system, we employed the offset parameter in our final update equation as well:
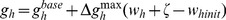
(46)


If the linear relationship between 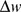 and 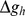 had unit slope, 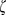 would be 0.25, and (46) reduced to 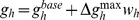, which was similar to the synaptic plasticity rule for updating AMPAR conductance based on *w* and the baseline value 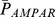 (Equations (9–11)). When the slope was not unity, and 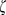 deviated from 0.25, the offset presented in (46) ensured that the update was consistent with the change in slope observed in Figure 4A. Thus, incorporating the offset parameter 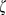 into equations (44) and (46) ensured that our rule was generalized across a range of baseline values of 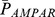 and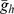, rather than having it work only for the case where the linear relationship between 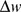 and 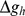 had unit slope.

Finally, our computation of 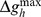 was from Figure 4A, which was obtained employing the IPR. We wanted to make this computation independent of IPR. In doing this, we searched for a function that reflected the sensitivities of *h*-channel plasticity to underlying parameters (Fig. S1A). Such a function would enable us to arrive at the relationship between 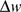 and 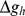 (Fig. 4A) without employing IPR. We empirically found that the function 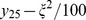 (Fig. S1F; 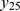 is the FF for 25 Hz SF, and 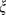 was as in (15)) reflected the sensitivities portrayed in (Fig. S1A), over a large range of underlying parameters. We employed this function to arrive at 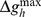 through an optimization procedure to match it with the sensitivity analysis presented for IPR (Fig. S1A):
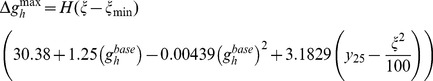
(47)where 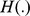 was the Heaviside function, that ensured that no plasticity occurred if the mean square error 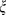 (Equation 15) was lesser than a minimum threshold value, 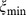. Once 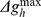 was obtained for a given set of parameters, the updated value of 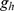 was computed from (41–46), as previously.

### Computation of Mutual Information

Mutual information between the output spikes and the input stimulus was employed as a measure of encoding capabilities of a neuron, and was computed as the difference between the total response entropy and the noise entropy [58], [59]:
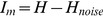
(48)where 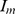 is the mutual information, 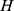 is the total response entropy, a measure of response variability and 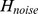 is the noise entropy. 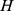 was calculated as:
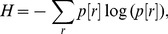
(49)where 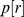 is the response probability distribution of a firing rate represented by 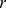, over the entire range of input stimulus frequencies. The input stimulus frequency, 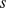, fed to the synapse was varied from 5 to 25 Hz in the steps of 1 Hz. The input spike timings were Poisson distributed, thus providing variability in response frequency for the same stimulus frequency, and the distribution of response frequencies for a given stimulus frequency 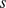 was represented as 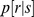. Each stimulus frequency was presented for 900 trials (each of 1 s duration) and the firing rate for each trial was calculated from the response of the neuron. For each stimulus frequency, 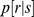 was then plotted from the first and second order statistics of these responses across trials, with an implicit assumption of a normal distribution for 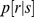 (Fig. 6A). This procedure was repeated for each stimulus frequency (Fig. 6A). The response probability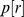, for each response frequency 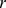, was then computed as (Fig. 6E):



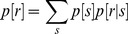
(50)In computing this, we considered 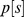 to be uniformly distributed as the presentation of different stimulus frequencies was equally probable. Plugging 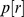 into equation (49) yielded the response entropy. To compute the noise entropy, we first computed the entropy of the responses for a given stimulus, 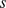, as:
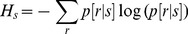
(51)


Noise entropy was then computed as:
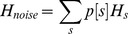
(52)


We computed mutual information by inserting the computed values of response and noise entropies into equation (48).

### Other Measurements

The synaptic drive to the model neuron was modeled as Poisson-distributed pre-synaptic action potentials arriving at various stimulus frequencies (SF). We presented the model with 100 trials of inputs at each SF and measured the corresponding firing frequencies (FF; Fig. 1C) to construct the input-output relationship of the model, when the neuron rested at –65 mV. We fixed the membrane potential in order to measure the effect of synaptic and/or *h*-channel plasticity on the FF-SF curve, without altering driving forces and/or the voltage-dependent conductance of all the channels present in the model [24], [25], [26], [32]. Each trial was made of synaptically driving the neuron with the specific SF for a 1 s period, and FF was computed by counting the number of action potentials in this 1 s period. FF for each SF was represented as mean ± SEM, computed over the 100 trails for that SF (Fig. 1C). It should be noted that the maximum SF used in arriving at this FF-SF curve was dependent on the specific choices of the underlying parameters, especially the baseline values of AMPAR permeability and *h* conductance, the two parameters that underwent plasticity. This was to ensure the sigmoidal parametric form for the FF-SF curve, which would be lost if firing entered the high frequency regime where it would be limited by the refractory period or depolarization-induced inactivation of the fast Na^+^ channels.

Firing frequencies in response to non-synaptic pulse current injections (*e.g.,* Fig. 2E–F) was determined by injecting a pulse current of various amplitudes into the neuronal model, and computing the number of action potentials fired per second.

## Supporting Information

Figure S1
**Sensitivity analysis for the iterative plasticity rule.** (**A**) Plot depicting the amount of change required in *h-*conductance to achieve firing rate homeostasis at various permeability values and baseline *h-*conductance values for the maximum synaptic perturbation (25 Hz/900 pulses LTP). (**B–D**) FF-SF plot for the parameter values indicated by the arrow B (**B**), C (**C**) and D (**D**) in panel (**A**). Black: Baseline; Red: after LTP inducation; Green: after LTP induction and IPR. At lower values of baseline 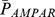, LTP induces a large shift in the FF-SF curve, thus requiring a larger change in *h*-conductance to compensate for the change (**B**). At intermediate values of baseline 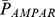, although the amount of change in FF-SF is small, the amount of change required in *h*-conductance was large because the baseline activity levels are higher (**C**; compare black traces in **B** and **C**). At larger values of baseline 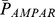, the amount of change in FF-SF curve was very minimal after LTP, thus, there was no plasticity in *h* conductance required (**D**). (**E**) The offset correction factor 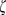 plotted for various values of baseline 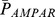 and *h* conductance. (**F**) To quantify this variability depicted in (**A**–**D**), we employed the term 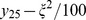, which represented the “competition” between activity of cell at the maximum stimulus frequency 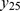 and the mean square error between the baseline and post-LTP FF-SF curves. This, in conjunction with terms involving baseline *h* conductance quantified the variability shown in (**A**–**D**) across different values of baseline 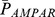 and *h* conductance.(TIF)

Figure S2
**Sensitivity analysis for the calcium-dependent plasticity rule (CDPR), also demonstrating changes in excitability obtained after CDPR.** (**A**) Plot depicting the effectiveness of CDPR in achieving firing rate homeostasis after synaptic plasticity induced with 900 pulses of different induction frequencies. As CDPR runs in parallel to synaptic plasticity, homeostatic gain control runs in parallel, reducing the root mean squared error (RMSE) between the achieved FF-SF curve and the target FF-SF curve below 1 Hz across all induction frequencies. (**B**) 3-D plot showing the updated *h-*conductance for various AMPAR permeability and baseline *h*-conductance values obtained by implementing CDPR for *h* channel plasticity in parallel with synaptic plasticity. Note that the sensitivity analysis presented here for CDPR is over a range smaller than the one presented for IPR. This was because the FF-SF lost its sigmoidal characteristic at larger AMPAR permeabilities and/or lower baseline 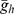. (**C**) Traces showing neuronal firing for a non-synaptic pulse current injection, before (black) and after LTP and CDPR (green). (**D**) Firing frequencies for various amplitudes of non-synaptic pulse current injections (of 500 ms) shown for cases before (black) and after LTP and CDPR (green). (**E**–**F**) Same as (**C**)–(**D**), but for LTD, when implemented in parallel with CDPR.(TIF)

Figure S3
**Sensitivity analysis for the information transfer across the neuron.** (**A**) Impact of repeated LTP (20.15 Hz/900 pulses, each induction) on AMPAR permeability depicted as a function of the number of LTP inductions, plotted for cases where synaptic plasticity was accompanied (green) and not accompanied (black) by CDPR. 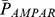 = 25 nm/s and baseline 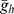 = 0.25 mS/cm^2^. (**B**) Mutual information plotted as a function of number of successive LTP runs, under cases where CDPR accompanied (green) or did not accompany (black) synaptic plasticity for parametric values as in (**A)**. (**C**) Impact of repeated LTP (13.1 Hz/900 pulses, each induction) on AMPAR permeability depicted as a function of the number of LTP inductions, plotted for cases where synaptic plasticity was accompanied (green) and not accompanied (black) by CDPR. 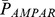 = 36 nm/s and baseline 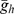 = 0.35 mS/cm^2^. (**D**) Mutual information plotted as a function of number of successive LTP runs, under cases where CDPR accompanied (green) or did not accompany (black) synaptic plasticity for parametric values as in (**C)**. (**E**) Impact of repeated LTP (10.55 Hz/900 pulses, each induction) on AMPAR permeability depicted as a function of the number of LTP inductions, plotted for cases where synaptic plasticity was accompanied (green) and not accompanied (black) by CDPR. 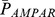 = 45 nm/s and baseline 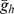 = 0.55 mS/cm^2^. (**F**) Mutual information plotted as a function of number of successive LTP runs, under cases where CDPR accompanied (green) or did not accompany (black) synaptic plasticity for parametric values as in (**E)**. The frequencies employed for induction are the sliding threshold frequencies for the corresponding baseline parameters of 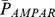 and 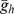.(TIF)
