# Supplementary figures and images for: A Calcium-Dependent Plasticity Rule for HCN Channels Maintains Activity Homeostasis and Stable Synaptic Learning
Source: PLoS One. 2013 Feb 4;8(2):e55590. doi: 10.1371/journal.pone.0055590 (PMC3563588; doi:10.1371/journal.pone.0055590)

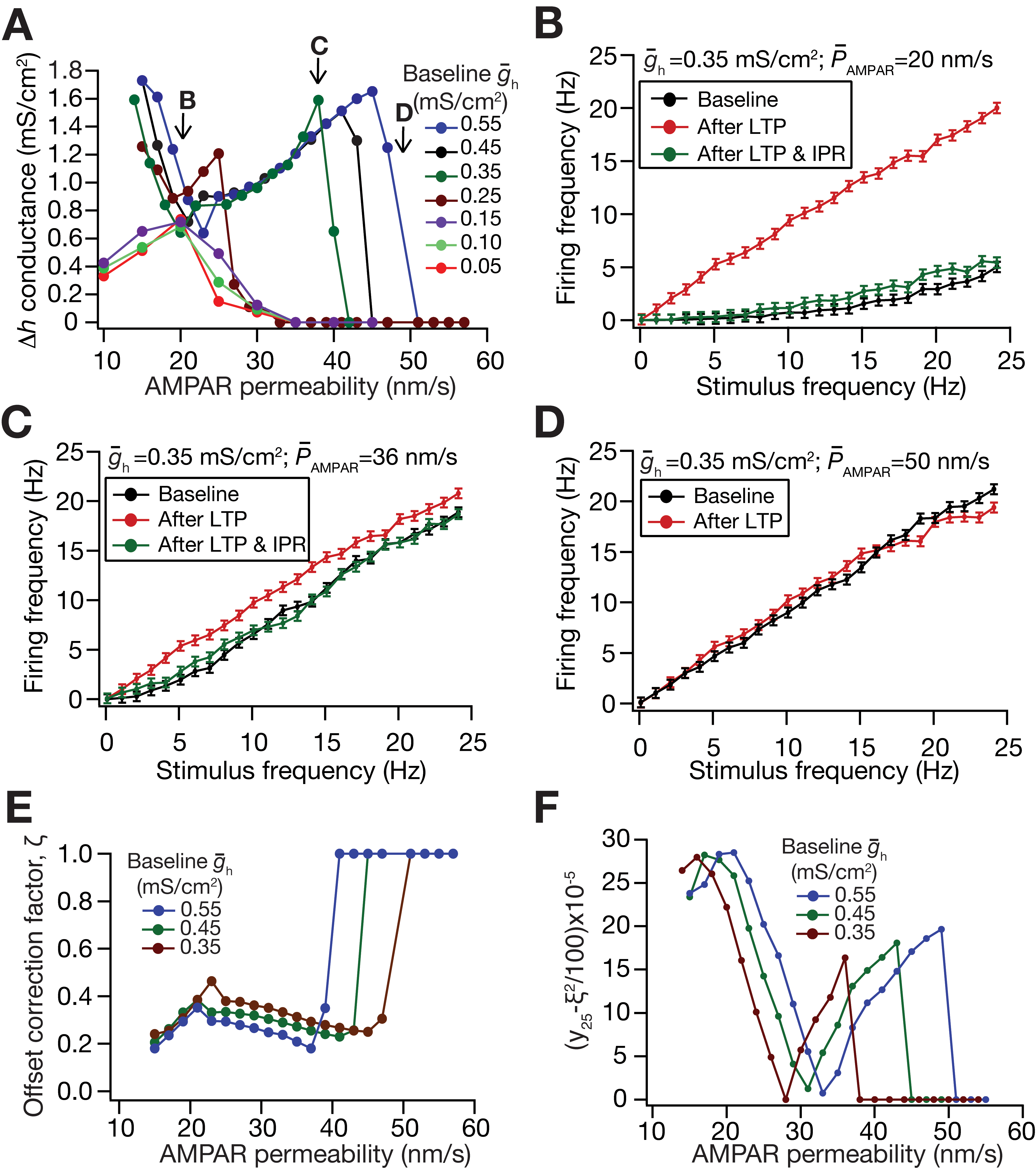

Supplement: Figure S1 — Sensitivity analysis for the iterative plasticity rule. (A) Plot depicting the amount of change required in h-conductance to achieve firing rate homeostasis at various permeability values and baseline h-conductance values for the maximum synaptic perturbation (25 Hz/900 pulses LTP). (B–D) FF-SF plot for the parameter values indicated by the arrow B (B), C (C) and D (D) in panel (A). Black: Baseline; Red: after LTP inducation; Green: after LTP induction and IPR. At lower values of baseline , LTP induces a large shift in the FF-SF curve, thus requiring a larger change in h-conductance to compensate for the change (B). At intermediate values of baseline , although the amount of change in FF-SF is small, the amount of change required in h-conductance was large because the baseline activity levels are higher (C; compare black traces in B and C). At larger values of baseline , the amount of change in FF-SF curve was very minimal after LTP, thus, there was no plasticity in h conductance required (D). (E) The offset correction factor plotted for various values of baseline and h conductance. (F) To quantify this variability depicted in (A–D), we employed the term , which represented the “competition” between activity of cell at the maximum stimulus frequency and the mean square error between the baseline and post-LTP FF-SF curves. This, in conjunction with terms involving baseline h conductance quantified the variability shown in (A–D) across different values of baseline and h conductance. (TIF) [file pone.0055590.s001.tif]

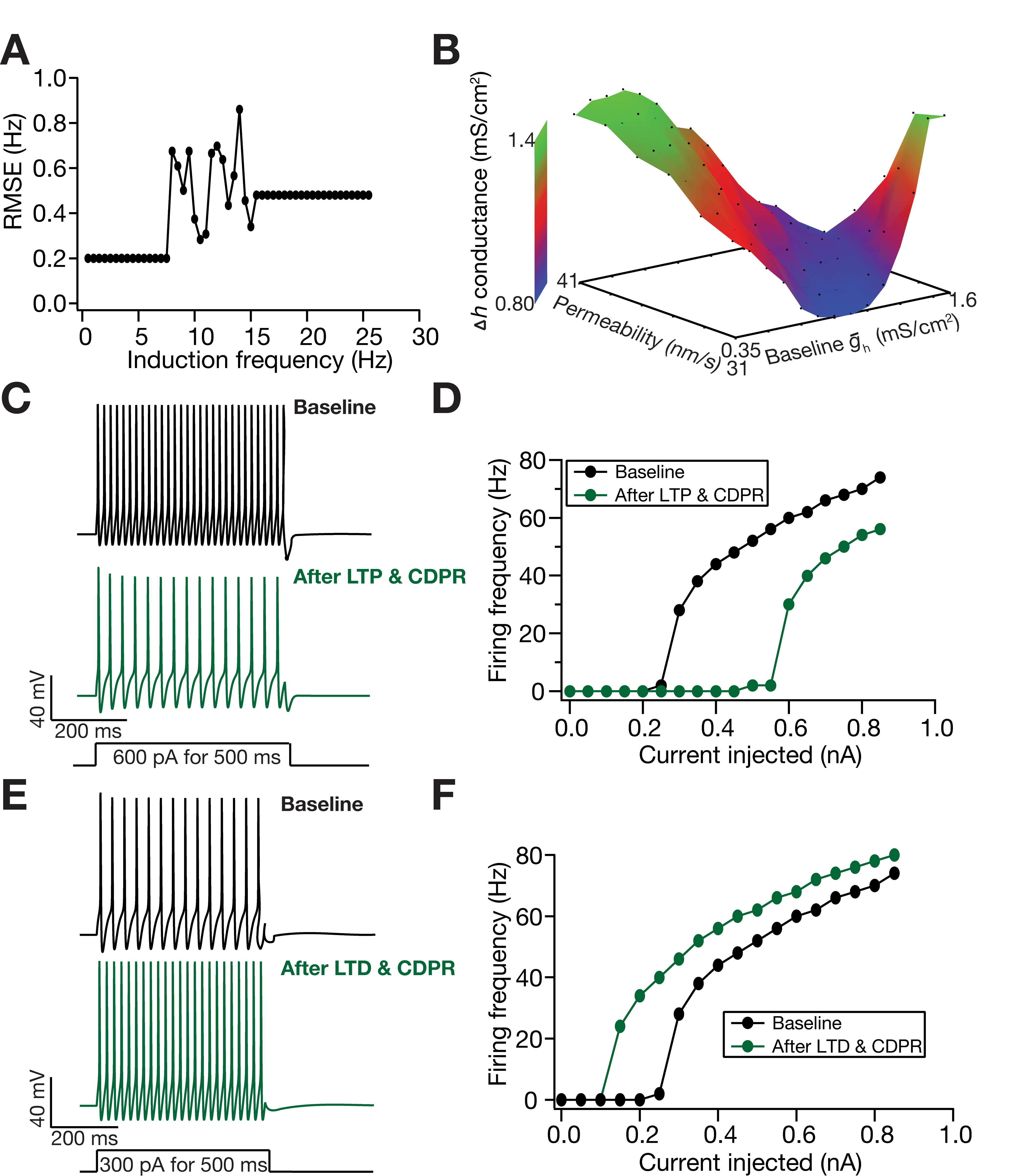

Supplement: Figure S2 — Sensitivity analysis for the calcium-dependent plasticity rule (CDPR), also demonstrating changes in excitability obtained after CDPR. (A) Plot depicting the effectiveness of CDPR in achieving firing rate homeostasis after synaptic plasticity induced with 900 pulses of different induction frequencies. As CDPR runs in parallel to synaptic plasticity, homeostatic gain control runs in parallel, reducing the root mean squared error (RMSE) between the achieved FF-SF curve and the target FF-SF curve below 1 Hz across all induction frequencies. (B) 3-D plot showing the updated h-conductance for various AMPAR permeability and baseline h-conductance values obtained by implementing CDPR for h channel plasticity in parallel with synaptic plasticity. Note that the sensitivity analysis presented here for CDPR is over a range smaller than the one presented for IPR. This was because the FF-SF lost its sigmoidal characteristic at larger AMPAR permeabilities and/or lower baseline . (C) Traces showing neuronal firing for a non-synaptic pulse current injection, before (black) and after LTP and CDPR (green). (D) Firing frequencies for various amplitudes of non-synaptic pulse current injections (of 500 ms) shown for cases before (black) and after LTP and CDPR (green). (E–F) Same as (C)–(D), but for LTD, when implemented in parallel with CDPR. (TIF) [file pone.0055590.s002.tif]

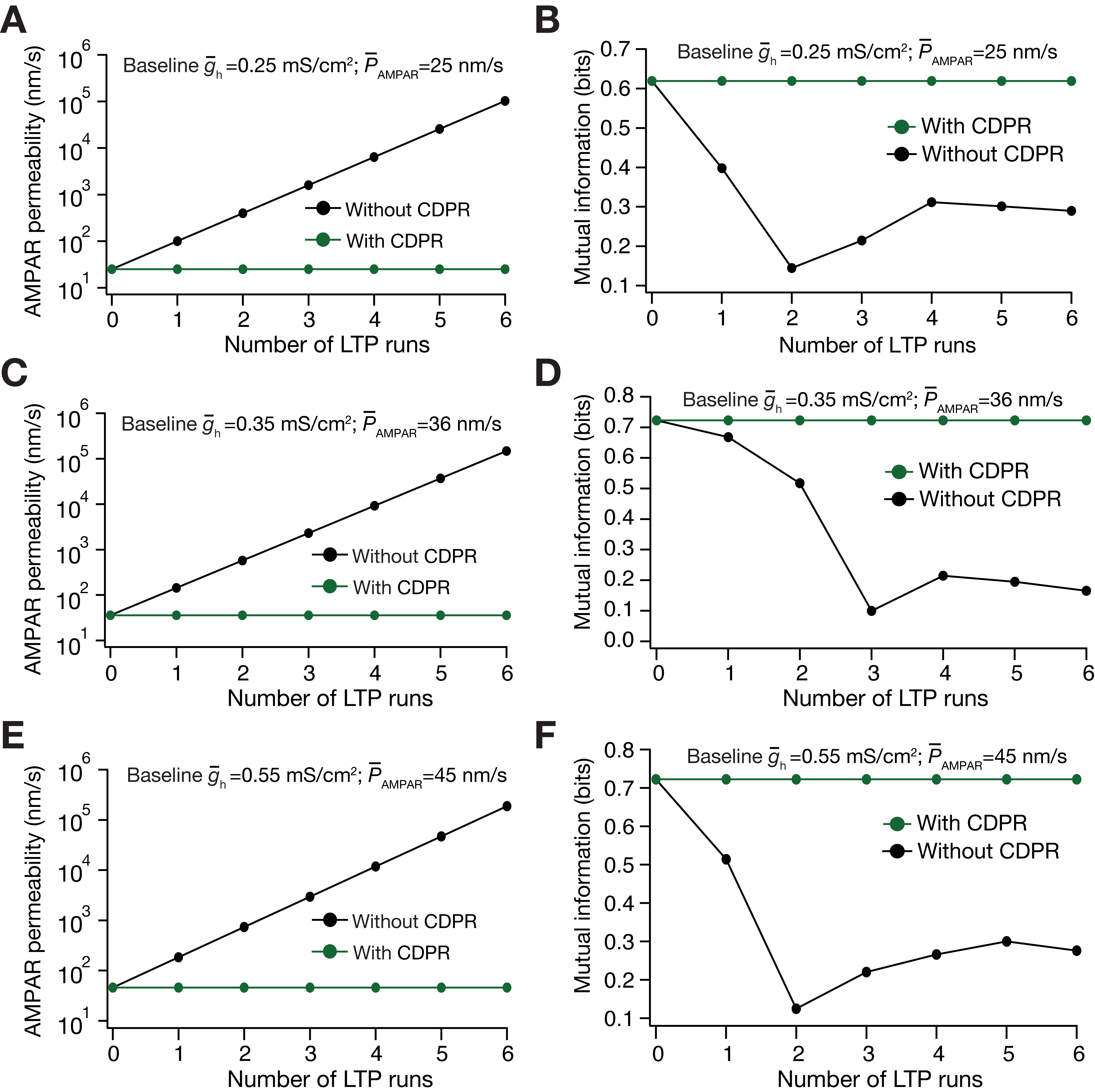

Supplement: Figure S3 — Sensitivity analysis for the information transfer across the neuron. (A) Impact of repeated LTP (20.15 Hz/900 pulses, each induction) on AMPAR permeability depicted as a function of the number of LTP inductions, plotted for cases where synaptic plasticity was accompanied (green) and not accompanied (black) by CDPR. = 25 nm/s and baseline = 0.25 mS/cm2. (B) Mutual information plotted as a function of number of successive LTP runs, under cases where CDPR accompanied (green) or did not accompany (black) synaptic plasticity for parametric values as in (A). (C) Impact of repeated LTP (13.1 Hz/900 pulses, each induction) on AMPAR permeability depicted as a function of the number of LTP inductions, plotted for cases where synaptic plasticity was accompanied (green) and not accompanied (black) by CDPR. = 36 nm/s and baseline = 0.35 mS/cm2. (D) Mutual information plotted as a function of number of successive LTP runs, under cases where CDPR accompanied (green) or did not accompany (black) synaptic plasticity for parametric values as in (C). (E) Impact of repeated LTP (10.55 Hz/900 pulses, each induction) on AMPAR permeability depicted as a function of the number of LTP inductions, plotted for cases where synaptic plasticity was accompanied (green) and not accompanied (black) by CDPR. = 45 nm/s and baseline = 0.55 mS/cm2. (F) Mutual information plotted as a function of number of successive LTP runs, under cases where CDPR accompanied (green) or did not accompany (black) synaptic plasticity for parametric values as in (E). The frequencies employed for induction are the sliding threshold frequencies for the corresponding baseline parameters of and . (TIF) [file pone.0055590.s003.tif]
